# Autotransplantation of a Supernumerary Tooth to Replace a Misaligned Incisor with Abnormal Dimensions and Morphology: 2-Year Follow-Up

**DOI:** 10.1155/2013/146343

**Published:** 2013-02-11

**Authors:** R. Ebru Tirali, Cagla Sar, Ufuk Ates, Metin Kizilkaya, S. Burcak Cehreli

**Affiliations:** ^1^Department of Pediatric Dentistry, Faculty of Dentistry, Baskent University, 11. Sokak No. 26, Bahcelievler, 06490 Ankara, Turkey; ^2^Department of Orthodontics, Faculty of Dentistry, Baskent University, Ankara, Turkey; ^3^Department of Oral and Maxillofacial Surgery, Faculty of Dentistry, Baskent University, Ankara, Turkey; ^4^Private Practice, 34100 Istanbul, Turkey

## Abstract

Autotransplantation is a viable treatment option to restore esthetics and function impaired by abnormally shaped teeth when a suitable donors tooth is available. This paper describes the autotransplantation and 2-year follow-up of a supernumerary maxillary incisor as a replacement to a misaligned maxillary incisor with abnormal crown morphology and size. The supernumerary incisor was immediately autotransplanted into the extraction site of the large incisor and was stabilized with a bonded semirigid splint for 2 weeks. Fixed orthodontic therapy was initiated 3 months after autotransplantation. Ideal alignment of the incisors was accomplished after 6 months along with radiographic evidence of apical closure and osseous/periodontal regeneration. In autogenous tooth transplantation, a successful clinical outcome can be achieved if the cases are selected and treated properly.

## 1. Introduction

Autotransplantation (autogenous tooth transplantation) refers to the repositioning of autogenous teeth in another tooth extraction site or a surgically prepared recipient site to replace teeth that are congenitally missing or have poor prognosis [1, 2]. Traumatic injuries affecting anterior teeth which require prosthodontic or orthodontic treatment may also be treated by autotransplantation [1–4]. Finally, autotransplantation can be utilized to replace teeth with shape/size anomalies using donor teeth of normal size and/or morphology [5]. 

The success of autotransplantation can be influenced by a number of factors, which include patient age, developmental stage of the transplanted tooth, type of tooth transplanted, surgical technique employed, and extra alveolar time span before the tooth is transplanted [1–4]. Root resorption and loss of attachment are the major complications of autogenous tooth transplants. Contraindications for autotransplantation include cardiac anomalies, poor oral hygiene, and poor self-motivation [3].

Autotransplanted teeth result in the maintenance and regeneration of alveolar bone, and the procedure can be performed in growing patients. The aim of this paper is to present the autotransplantation, orthodontic treatment, and 2-year follow-up of a supernumerary incisor as a replacement to a large, fused central incisor. 

## 2. Case Presentation

**Figure 1:**
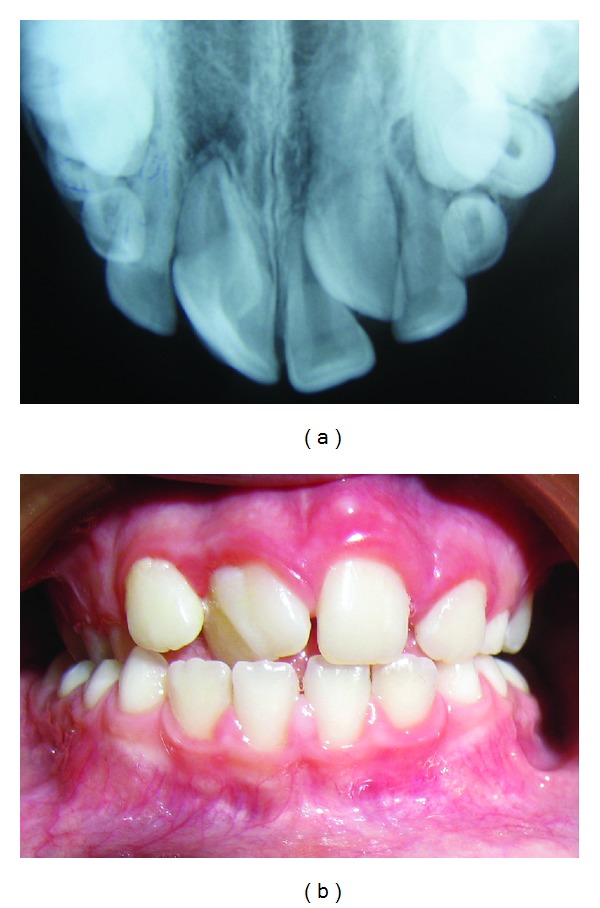
(a) Occlusal radiograph of the patient, demonstrating the large incisor and the unerupted supernumerary incisor. (b) Close-up view of the tooth, showing abnormal crown dimensions and morphology.

A healthy, 10-year-old boy was admitted to pediatric dentistry clinic for the management of crowding associated with a large tooth present in the upper jaw. On intraoral examination, the patient's chief complaint was confirmed by the presence of a misaligned maxillary right central incisor. The tooth had a talon cusp-like enamel projection on the labial aspect of the crown and had a large mesiodistal dimension. Owing to the patient's gagging reflex, a proper radiographic examination of the incisor could not be made. However, the occlusal and periapical radiographs were highly suggestive of a large pulp chamber, and even of the possibility of a fused tooth. The occlusal radiograph also indicated an unerupted supernumerary incisor located on the contralateral side between the central and lateral incisors. The supernumerary incisor had a well-shaped crown and showed advanced root formation with incomplete apical closure (Figure 1). 

**Figure 2:**
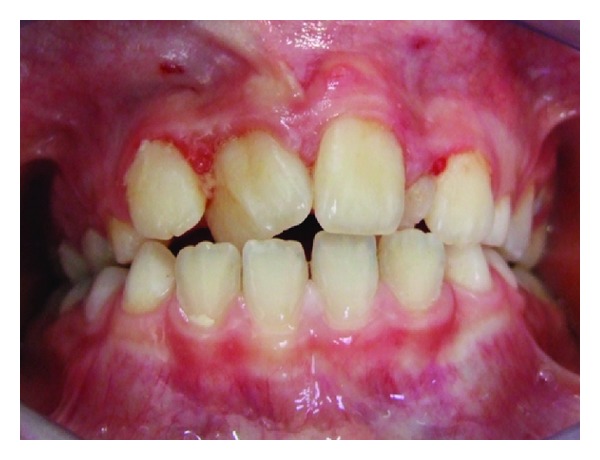
View of the supernumerary incisor (arrow), which erupted 4 months after the initial examination.

**Figure 3:**
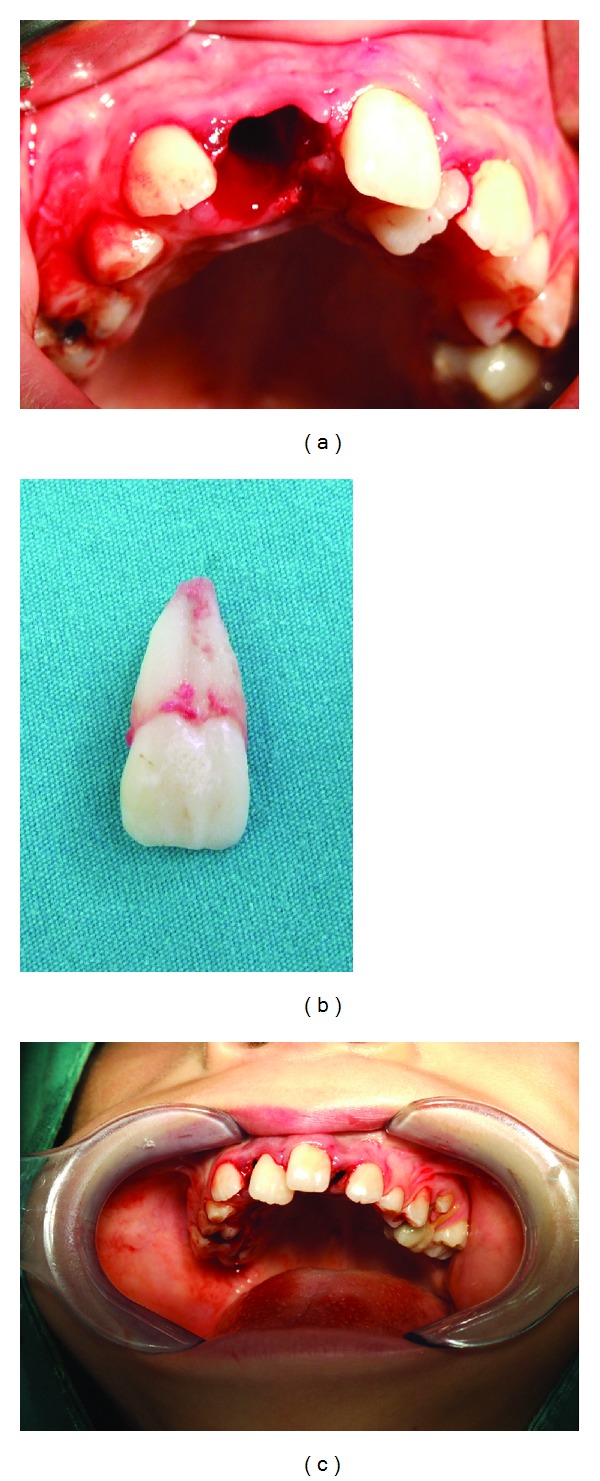
(a) View of the extraction socket of the large incisor; (b) the extracted incisor, demonstrating large crown and root dimensions and morphological appearance of a fused tooth; (c) view of the supernumerary incisor, immediately after implantation into the recipient site.

**Figure 4:**
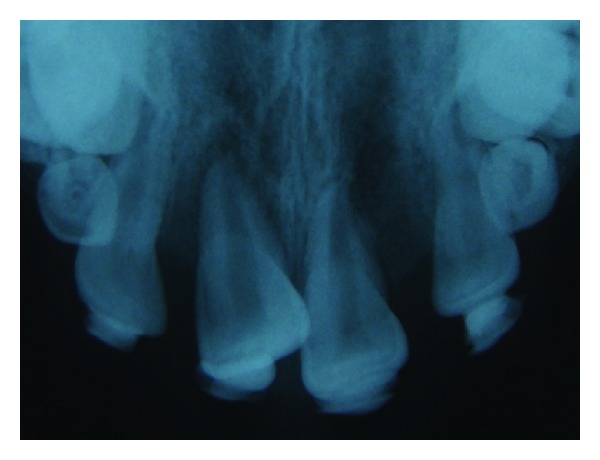
Radiographic view of the autotransplanted incisor at the 3rd week.

**Figure 5:**
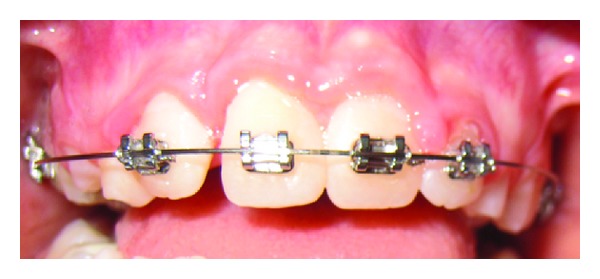
View of the incisors during fixed orthodontic treatment.

**Figure 6:**
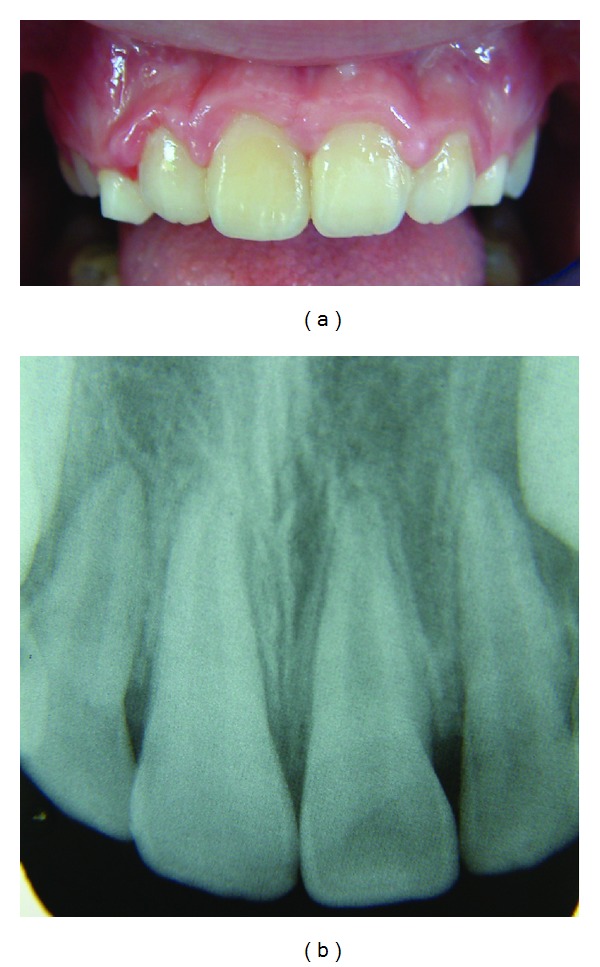
Clinical and radiographic view of the maxillary incisor at 2 years.

Among possible treatment options, esthetic reduction of the crown in both the proximal and labial aspects was considered unfavorable due to the presence of a large pulp chamber. Extraction of the tooth and consequent orthodontic correction were also discarded as a treatment alternative, since orthodontic movement of the supernumerary tooth over the midline could result in resorption of the alveolar bone. Finally, the use of the contralateral supernumerary incisor as a replacement for the existing large incisor was considered the best treatment option, since both the radiographic crown dimensions and presence of the immature apex favored the possibility for autotransplantation. The patient and his parents were informed about the treatment options and their possible outcomes. The patient showed up four months later, approving the treatment plan of autotransplantation. During the time, the supernumerary incisor erupted into the crowded maxillary arch in a rotated fashion (Figure 2). Extraction of the large incisor and autotransplantation of the supernumerary tooth were made one week later under local anesthesia (Figure 3). Stabilization of the autotransplanted incisor was provided by use of a semirigid splint made of 0.9 mm fisherman spring, bonded with acid-etch composite resin. The gingival tissue on the mesial and distal aspects of the tooth crown were sutured to accelerate healing. The patient was prescribed 20 mg/kg amoxicillin two times a day and a chlorhexidine gluconate mouthrinse for 1 week along with strict hygiene instructions. The splint was removed at the end of the second week, and the mobility was observed to be within normal limits. The radiographic followup demonstrated initiation of periradicular healing in the absence of clinical symptoms and pathologic mobility (Figure 4). Orthodontic treatment was initiated 3 months after autotransplantation and the transplanted tooth had an acceptable gingival contour, showed normal mobility, and responded to thermal and electrical pulp tests at the beginning of fixed orthodontic treatment (Figure 5). Clinical and radiographic followup at 2 years demonstrated apical closure, advanced regeneration of the periapical tissues, and reestablishment of the periodontal space along with slight calcific metamorphosis of the root canal space (Figure 6). 

## 3. Discussion

Esthetic management of anterior teeth with abnormal crown dimensions and/or morphology may be quite challenging. As seen herein, the large coronal pulp space may limit the chance of successful reduction of the crown dimensions without possible endodontic complications. In such cases, autotransplantation appears to be a viable treatment option to restore esthetics and function, especially when a suitable donor tooth is available [1–5]. In young individuals, successful tooth transplantation also facilitates dentofacial development, mastication, and speech along with maintenance of the attached gingiva with a natural shape and level [1–4]. 

High long-term success rates of autotransplantation have been reported in the dental literature [6–8]. The age of the patient, the type, and development stage of the donor tooth are important factors that affect the success of autotransplantation [6, 8]. Further, a healthy periodontal membrane should be present on the transplanted tooth and the root morphology of the tooth to be transplanted should be simple. In addition, infection should be absent in the recipient site, and during surgery, the extraoral period should be short and trauma should be minimized [9–11]. 

As observed in the present case, calcific metamorphosis of the pulp is a common finding in transplanted teeth [5, 12–14]. No endodontic treatment was made since the pulp was vital and asymptomatic. According to Andreasen and Hjorting-Hansen [12], endodontic treatment of such teeth should be started only when a tooth becomes symptomatic or when bone lesions develop. Autotransplanted teeth have been shown to serve without pulpal complications for many years. 

The presence of intact and viable periodontal ligament cells on the root surface of the donor tooth is the most critical factor that determines the prognosis of an autotransplanted tooth [1, 3, 11, 15]. Extended extraoral time of the donor tooth significantly affects the viability of the periodontal ligament cells, which leads to unfavorable results such as inflammation or root resorption [4]. In the present case, the supernumerary (donor) incisor was extracted after the fused incisor in order to minimize the extraoral dry time. The autotransplantation was performed immediately thereafter, without immersing the tooth in any kind of transport/storage media. 

Along with rapid and atraumatic surgical technique, adequate recipient site affects the prognosis as well [15, 16]. Unlike other organ transplants, tooth transplants require dimensional compatibility between the transplanted tooth and the recipient site [17]. Optimal contact with the recipient site can improve the level of nutrition and the blood supply to the periodontal ligament cells, which can increase the success rate of autotransplantation [3, 18, 19]. In the absence of adequate buccolingual width to accommodate the donor tooth, resorption of the alveolar ridge may occur [18, 19]. In the present case, the buccolingual dimension of the donor tooth was compatible with the width of the transplantation socket, but the mesiodistal size of the donor tooth was considerably smaller. Thus, in order to maximize adaptability, the donor tooth was placed in a rotated fashion and gingival tissue on the mesial and distal aspects of the tooth crown were sutured to optimize postoperative healing. This procedure proved out to be effective, as evidenced by the final clinical and radiographic outcome.

Various techniques have been described to stabilize transplanted teeth, including loose fixation with sutures, ligatures, orthodontic brackets, acid-etch composite and wire splints, and ligature wires or orthodontic appliances [20–22]. The reported duration of splinting varies between 1 week [18, 19] and 4–6 weeks [23, 24]. In selected cases, splinting may not even be necessary [6]. It has been shown that prolonged and rigid fixation has significant negative influence on the success of tooth transplantation [25–27]. Thus, a short-term (2-week) semi-rigid splint was used in the present case.

 Transplantation of teeth with immature roots offers high success rates due to the chance of revascularization as well as unimpeded development of the donor tooth and adjacent alveolar bone growth [5, 12, 16, 18]. Transplanted teeth with incomplete root formation possess a 96% rate of pulpal revascularization, while those with complete root formation show considerably lower chances (i.e., about 15%) of pulp regeneration [22, 28]. In the present case, the donor tooth showed advanced root formation, with incomplete apical closure. Since pulpal vitality was maintained after transplantation, no endodontic intervention was performed. 

 Transplanted teeth can be submitted to orthodontic treatment 3 to 6 months after transplantation [6, 10, 18, 29, 30]. According to Hamamoto et al. [31], orthodontic treatment can be initiated after regeneration of the periodontal space and subsequent radiographic confirmation of the lamina dura. In the present case, tooth alignment was accomplished with a short-term orthodontic intervention which was initiated approximately 4 months following autotransplantation. During the first month of orthodontic treatment, the amount of the force was minimized by leaving the autotransplanted tooth outside of the archwire. 

The outcome of autotransplantation can be considered successful if there is no progressive root resorption, the adjacent periodontal tissues adjacent are normal and the crown-to-root ratio is less than 1 [7, 22]. In the present case, the success of autotransplantation also complied with the modified criteria of Chamberlin and Goerig [32]. Accordingly, the tooth was fixed in its socket without discomfort; the patient was chewing satisfactorily and without discomfort; the tooth was not mobile; no pathological condition was seen on the radiograph; the lamina dura appeared normal on the radiographs; and the depths of the sulcus, gingival contour, and gingival colour were normal. In addition, the crown to-root-ratio was less than 1. At the follow-up appointments, no pulpal or periodontal complications were observed.

## 4. Conclusion

In the presence of a well-shaped supplementary tooth, autotransplantation may be considered as a viable treatment option for replacement of large, misaligned teeth, which impair both esthetics and function. The procedure is cost-effective, one-stage surgery can be used and orthodontic movement is possible. Hence, autotransplantation can be considered as a successful and atraumatic treatment option in growing patients, especially when compared with treatment alternatives such as interim removable prostheses.
